# The action of selection on codon bias in the human genome is related to frequency, complexity, and chronology of amino acids

**DOI:** 10.1186/1471-2164-7-67

**Published:** 2006-04-03

**Authors:** Daniel Kotlar, Yizhar Lavner

**Affiliations:** 1 Department of Computer Science, Tel-Hai Academic College, Upper Galilee, 12210, Israel

## Abstract

**Background:**

The question of whether synonymous codon choice is affected by cellular tRNA abundance has been positively answered in many organisms. In some recent works, concerning the human genome, this relation has been studied, but no conclusive answers have been found. In the human genome, the variation in base composition and the absence of cellular tRNA count data makes the study of the question more complicated. In this work we study the relation between codon choice and tRNA abundance in the human genome by correcting relative codon usage for background base composition and using a measure based on tRNA-gene copy numbers as a rough estimate of tRNA abundance.

**Results:**

We term *major codons *to be those codons with a relatively large tRNA-gene copy number for their corresponding amino acid. We use two measures of expression: *breadth of expression *(the number of tissues in which a gene was expressed) and *maximum expression level among tissues *(the highest value of expression of a gene among tissues). We show that for half the amino acids in the study (8 of 16) the *relative major codon usage *rises with breadth of expression. We show that these amino acids are significantly more frequent, are smaller and simpler, and are more ancient than the rest of the amino acids. Similar, although weaker, results were obtained for maximum expression level.

**Conclusion:**

There is evidence that codon bias in the human genome is related to selection, although the selection forces acting on codon bias may not be straightforward and may be different for different amino acids. We suggest that, in the first group of amino acids, selection acts to enhance translation efficiency in highly expressed genes by preferring major codons, and acts to reduce translation rate in lowly expressed genes by preferring non-major ones. In the second group of amino acids other selection forces, such as reducing misincorporation rate of expensive amino acids, in terms of their size/complexity, may be in action.

The fact that codon usage is more strongly related to breadth of expression than to maximum expression level supports the notion, presented in a recent study, that codon choice may be related to the tRNA abundance in the tissue in which a gene is expressed.

## Background

The question of whether codon bias, the unequal use of synonymous codons [1,2] is acted upon by selection has been studied extensively in many organisms, including Homo sapiens. One of the models explaining the relations between codon bias and selection is the selection for translation efficiency model (or the translation-selection model): codon usage in highly expressed genes is biased toward "optimal" codons corresponding to the more abundant tRNAs. This affects both elongation rate and accuracy [3,4]. Clear evidence for this model was found in prokaryotes such as E. coli [5], but not in all bacteria [6]. In addition, [7] showed for E.coli, that different codons, coding for the same amino acid, differ in their sensitivity to amino acid starvation (as determined by the level of the corresponding charged tRNA isoacceptor) thus accounting for different codon choice. Support for the translation-selection model was also found in some eukaryotes: S. Cerevisiae [8-11], C. elegans [12,13] and Drosophila [10,14], and even vertebrates: Xenopus laevis [15]. In contrast, in the human genome, the evidence is less clear. The Isochoric structure [16] of the human genome appears to be the most influential factor on codon composition, and has been shown to be related to expression level (see [17] among many studies). Thus, any attempt to unravel the relation between expression level and codon choice in the human genome has to control for the relation between background base composition and expression level. A few recent studies point at some evidence: Urrutia and Hurst [18,19] found a weak correlation between gene expression level and codon bias, but could not relate this correlation to tRNA abundance. Recently, Chamary and Hurst [20] have showed evidence that the action of selection on synonymous mutations in mammals is related to the stability of mRNA secondary structure. Other evidence for selection acting on synonymous codon choice, associated with splicing enhancers, which results in codon bias, is reported by Willie and Majewski [21], Chamary and Hurst [22], Fairbrother et al. [23], and Parmley et al. [24] (see also [1] for a review). In these studies the authors found preference for codons that are well-represented in exonic splicing enhancers (ESEs, [23]), and thus support the 'enhancer model' [21,22]. Comeron [25] showed that in the human genome, in the majority of amino acids with degeneracy greater than one, the codons with the most abundant tRNA gene copy numbers exhibit an increase in frequency in highly expressed genes compared to lowly expressed genes. Plotkin et al. [26] showed that codon usage in tissue specific genes varies among genes expressed in different tissues, suggesting that it could be affected by differential tRNA abundances. This variability of the codon usage among tissues was confirmed by Se'mon et al. [27]. However, using internal correspondence analysis, the latter authors showed that the variability of synonymous codon usage between tissues represents only 2.3% of the total codon usage variability, and that most of this is explained by isochore-scale variability of GC-content that affects both coding and introns or intergenic regions.

In a recent study [28], we showed a U-shape relation between codon bias and expression level, namely that codon bias is highest for both highly and lowly expressed genes, and that the frequency of optimal codons (FOP) rises with expression. We proposed two other ways in which selection may act on codon bias: regulating expression of lowly expressed genes by preferring codons with less tRNAs, and enhancing translation accuracy, by preferring optimal codons in genes whose corresponding proteins have a high concentration of "expensive" amino acids.

In this study we further examine the model proposed in [28] by looking at the relation between the RSCU' (relative synonymous codon usage, corrected for background nucleotide content) of each codon, and expression level in more than 16,000 human genes. We look at the major codons, namely the codons with a higher amount of tRNA genes for their amino acids, and look at the *relative major codon usage *(RMCU) of each amino acid and its relation to expression level and expression breadth. We show evidence for the translation-selection model in the smaller, more frequent, and presumably ancient amino acids. In the remaining amino acids, other, not completely understood, selection forces may be more dominant. These forces may include the enhancement of translation accuracy, by an increased frequency of codons corresponding to more abundant tRNAs, in genes that translate to proteins with a high concentration of expensive amino acids in terms of their size/complexity.

## Results

In [28] we showed that both highly expressed genes and lowly expressed genes are characterized by high codon bias. In order to show that the high codon bias in the highly expressed genes results from preference for different codons than in the lowly expressed genes, we studied the relation between the RSCU' (see Methods) of each codon and expression level, measured by breadth of expression (see Methods). For each codon, we compared the mean RSCU' of the genes whose breadth of expression is below the 25^th ^percentile, with the mean RSCU' of the genes whose breadth of expression is above the 75^th ^percentile. The same comparison has been carried out for maximum expression (see Methods). The results are listed in columns D and E of Table 1. The significance of the difference was determined after performing randomizations, as described in the Methods section. The entries of column E of Table 1 show that the RSCU' of most codons is either positively or negatively correlated with breadth of expression, indicating that indeed the high codon bias in the genes with low breadth of expression results from preference for different codons than in the genes with high breadth of expression. However, for maximum expression level, the RSCU' of most codons in the lowest quartile is not significantly different than it is in the highest quartile.

If selection is involved in shaping codon bias, we expect that the RSCU' of favored codons (in terms of translation efficiency) will be positively correlated with expression, while the RSCU' of non-favored codons will be negatively correlated, or uncorrelated, with expression. We use the tRNA-gene copy number to determine which codons are favored, as in [12,25,28]. The rationale for using tRNA-gene copy numbers for this purpose is explained in [12] and in [28]. The values of tRNA gene copy numbers refer to the human genome assembly of May 2004 (see Methods).

The data in Table 1 indicates that the RSCU' of major codons does not always rise with expression level (measured in either of the two ways: breath of expression or maximum expression), as we would expect by the translation-selection model. However, what really counts is not the relative frequency of single codons, but rather the combined frequency of all the major codons of a given amino acid. We use the relative major codon usage, or RMCU, defined in the Methods. We first discard from the analysis the amino acids that do not have major codons according to the definition in the Methods. Then, for each of the remaining amino acids, we study the relation between the RMCU and both measures of expression, among the genes where the amino acid has at least 10 appearances. The results are listed in Table 2. We compared the average RMCU in the lower and higher quartiles, in the same way it has been performed for the RSCU'. Differences were considered significant, after conducting randomizations, according to the rule described in the Methods. Amino acids of degeneracy 6 were split into two amino acids of degeneracies 2 and 4 for the sake of correction for background base composition, as described in the Methods.

**Figure 1:**
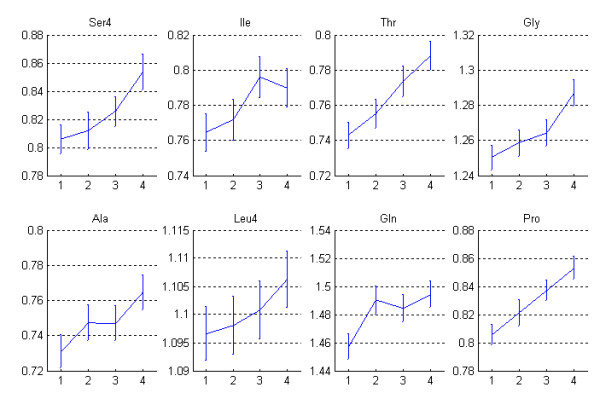
RMCU (vertical axis) of amino acids in Group A vs. breadth of expression. The bins on the horizontal axis represent breadth of expression as follows: bin 1: 1–5, bin 2: 6–9, bin 3: 10–13 and bin 4: 14–20.

**Figure 2:**
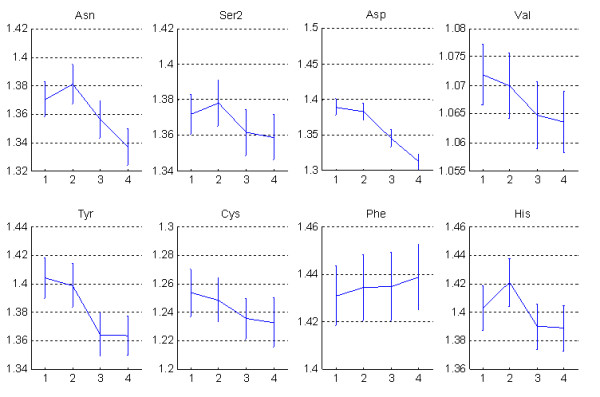
RMCU (vertical axis) of amino acids in Group B vs. breadth of expression. The bins on the horizontal axis represent breadth of expression as follows: bin 1: 1–5, bin 2: 6–9, bin 3: 10–13 and bin 4: 14–20.

As Table 2 reveals, for most amino acids there is no significant relation between the RMCU and maximum expression level. However, when breadth of expression is concerned, for eight of the 16 amino acids studied (Ala, Gln, Gly, Ile, Leu4, Pro, Ser4, Thr) their RMCU is in positive correlation with breadth of expression, as expected by the translation selection model. We denote this group of amino acids as Group A. For three amino acids (Asn, Asp, Tyr) their RMCU is in negative correlation with breadth of expression, apparently opposing the translation selection model, and for the remaining five there is no significant correlation, and we denote all the latter eight amino acids as Group B. (There are five amino acids not included in this analysis, as they do not have major codons, according to our definition). Figure 1 contains bar graphs describing the relation between breadth of expression and RMCU for the amino acids in Group A. (It is evident from Table 1 that for 7 out of the 8 amino acids from group A, the RSCU of the major codon is in positive relation with breath of expression). Figure 2 contains bar graphs describing the relation between breadth of expression and RMCU for the amino acids in Group B.

We look to see whether this partition into Groups A and B is related to other amino acid attributes. In Table 3 we sorted the amino acids according to frequency in the genome (columns A-C), size/complexity score (columns D-F), and amino acid chronology (columns G-I). Frequencies were calculated in the 16,627 genes expressed in the 53 SAGE libraries in this study. As a measure of biosynthetic cost (column F) we use the size/complexity score of Dufton [29]. In column I we used Trifonov's chronology ranking [30].

**Figure 3:**
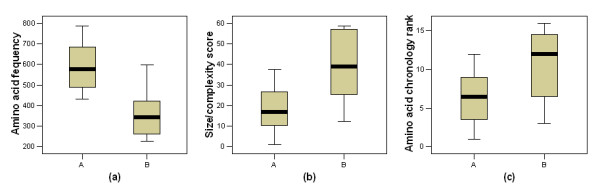
Frequency (a), Size/complexity score (b) and Chronology rank (c) of amino acids in Group A versus Group B. The bars designate the minimum and maximum values, the box designates the interquartile range, and the thick line in the middle represents the median.

The eight amino acids in Group A are among the ten most frequent amino acids that have major codons. Evidently, the amino acids in Group A are significantly more frequent than the ones in Group B (one-tailed Mann-Whitney test, p = 0.002). Similarly, the amino acids in Group A have significantly lower size/complexity score than the amino acids in Group B (one-tailed Mann-Whitney test, p = 0.015) and the amino acids in Group A are significantly more ancient than the amino acids in Group B (one-tailed Mann-Whitney test, p = 0.041). These properties are illustrated in the box-plots in Figure 3.

When we look at RMCU vs. maximum expression level (tags per 200,000) we see that only five amino acids (Ala, Gln, Gly, Ser4, and Leu4) exhibit an ascending relation, only one amino acid (Asp) exhibits a descending relation and the remaining ten amino acids show no ascending or descending relation between RMCU and maximum expression level. Defining Group A to contain the amino acids Ala, Gln, Gly, Ser4, and Leu4, and Group B as the set of the rest, we find again that the amino acids in Group A are significantly more frequent, have significantly lower size/complexity score, and are significantly more ancient than the amino acids in Group B (one-tailed Mann-Whitney test, p = 0.01, 0.032, 0.045 respectively).

Finally, we notice another set of codons, which we term the *translationally weak codons *– the non-major codons (in amino acids that have major codons), which do not translate through the wobble effect (marked with * in Table 1). There are nine such codons. For seven out of these nine codons the RSCU' is in negative correlation with both breadth of expression and maximum expression level, which is what is expected by the translation-selection model. The RSCU' of one translationally weak codon correlates negatively with maximum expression, but has no significant positive or negative correlation with breadth of expression, and there is only one exception (GTA coding for Valine).

## Discussion

In this paper we studied the translation-selection model in the human genome by looking at the relation between the RSCU' (RSCU corrected for background nucleotide content) of each codon and two measures of expression: maximum expression across tissues and breadth of expression, both based on SAGE data. We defined for each amino acid the *relative major codon usage *(RMCU), which is assumed to be a measure of tRNA availability, and thus of translation efficiency, based on the assumption that tRNA gene copy numbers are indicative of cellular tRNA abundance [12,25,28]. We studied the relation between the RMCU of amino acids that have major codons and the two measures of expression.

The results of this study can be summarized as follows:

1. Codon usage characterizing lowly expressed genes is different from the codon usage characterizing the highly expressed genes, although this difference is more apparent when expression is measured by breadth of expression rather than by expression level.

2. Amino acids whose RMCU rises with expression, in agreement with the translation selection model, are more frequent, less complex (in terms of size/complexity score, see [29]) and more ancient than those amino acids whose RMCU behavior does not agree with this model.

3. The above two results are much clearer for breath of expression than for maximum expression level.

These results suggest that codon bias in the human genome is related to selection, although the selection forces acting on codon bias may be different for different amino acids. As opposed to other studied organisms, where the tracks of selection are clearer and the presumed action of selection on codon bias is mainly to enhance translation efficiency as expression rises, in the human genome the situation seems to be more intricate: the signature of selection on codon bias is harder to detect and is possibly blurred by other stronger forces acting on codon bias.

Apparently, the results about the relation between RMCU and expression for the amino acids in Group A support the translation selection model in the human genome. Since these constitute most of the proteins produced (Figure 3a), it can be argued that the signature of selection is evident in the human genome. However, the amino acids in Group B, and the fact that the results are a lot less clear when dealing with maximum expression level compared to breadth of expression, cannot be overlooked.

As Figure 3b indicates, the amino acids not corresponding to Group A are more expensive than those in Group A (in terms of biosynthetic cost, as can be deduced from their size/complexity score), Since the price of misincorporation for expensive amino acids should be higher than for the cheap ones, we propose another force that may act on codon bias, namely, increasing the relative frequency of major codons to reduce misincorporation rate. This force may counteract selection for translation efficiency, especially when amino acids with high size/complexity score are concerned.

It should be noted that all amino acids in Group B, despite the fact that their RMCU does not rise with expression, have another codon, whose relative frequency rises with expression, which translates via the wobble effect, using tRNAs corresponding to major codons (Table 1, denoted with ^w^). Thus, although wobble pairing is less preferable to complete Watson and Crick pairing [9,31], more tRNAs are available for the codons of these amino acids as expression level rises. Thus, possibly other forces, as those mentioned above, are more dominant than the simple translation selection pressure for increased usage of major codons, as expression level rises.

As noted above, codon usage seems to be more related to the number of tissues in which a gene is expressed than to the maximum expression level in the different tissues. Hence, the synonymous codon usage is affected by how specific a gene is. It was shown [26] that codon usage differs between genes selectively expressed in different human tissues. This suggests that codon choice may be affected by the actual amounts of tRNA molecules in the tissue in which the gene is expressed. If the amounts of the different tRNAs vary between tissues, these amounts may have little to do with the tRNA gene copy numbers. Thus, any analysis based merely on tRNA gene copy numbers will fail to detect the effect of translation-selection, at least when it comes to genes of low breadth of expression. As far as we know, studies on cellular tRNA abundances in human tissues have not yet been performed, leaving us with tRNA gene copy numbers as the sole indicator of tRNA abundance. Such studies are essential for further understanding the role of selection in affecting codon choice in the human genome.

Another disadvantage of using tRNA gene copy numbers as indicators of tRNA abundance is that we can say nothing about amino acids for which the tRNA genes are distributed almost evenly among their codons. Such are Arginine, Lysine and Glutamine, and the 2-fold part of Leucine. As the numbers of tRNA genes vary slightly from one genome assembly to another, and since we assume a rough relation between tRNA gene copy numbers and tRNA abundances, small variation in tRNA gene copy numbers of synonymous codons have no meaning.

A caveat should be mentioned. Since both codon bias and expression measurements are affected by background composition, any result concerning codon bias and expression largely depends on how codon bias is corrected for background nucleotide content and on the way expression is measured. The results in Table 1 do not totally coincide with the results of [25]. This is attributed to two main factors: first, Comeron used microarray expression data and his analysis is tissue specific, while our expression data is est-SAGE, and we combine all available tissues. Second, the correction for background nucleotide content is different in the two works: while we corrected each RSCU value (see Methods), in [25] the uncorrected RSCU values were used in different gene classes, according to GC-composition. Nevertheless, the results in the two works indicate that there is great agreement between the class of codons whose RSCU rises with expression and the codons with the largest tRNA-gene copy number, and thus, support the notion of selection acting on codon bias.

Last, as mentioned in the introduction, there has recently been growing evidence that codon bias in exonic regions near junctions results from preference for certain codons, associated with splicing enhancers [21-24]. Since intron density is high in broadly expressed genes [25], the relation between expression level and codon usage may be explained by effects owing to splice regulation. To control for these effects, the analysis was repeated after removing the exonic regions near junctions (30 bp from each side). All the above results still remain, with the exception that the RMCU of Isoleucine no longer rises with expression. However, even when Isoleucine is discarded from Group A and added to Group B, the differences described in Figure 3 are still significant (one-tailed Mann-Whitney test, p = 0.001 (frequency), p = 0.042 (size/complexity score), and p = 0.027 (amino acid chronology rank). This indicates that the results obtained in our study are probably not related to splice regulation effects.

## Conclusion

In this whole genome analysis of the human genome, we showed that codon bias is related to selection, but in a more intricate way compared to mechanisms of selection in other organisms. We showed that different selection forces may shape codon bias for different amino acids. For the first group, namely the smaller, simpler, more abundant, and presumably ancient amino acids, the evidence supports the translation-selection model. We suggest that, in this group of amino acids, selection acts to enhance translation efficiency in highly expressed genes by preferring major codons, and acts to reduce translation rate in lowly expressed genes by preferring non-major ones. For the second group, which includes heavier and more complex amino acids, other mechanisms, such as reducing misincorporation rate of expensive amino acids, may be in action.

## Methods

### Sequence data

Gene and intron sequences were downloaded from NCBI GenBank build 35, updated on August 27, 2004 . We included only CDSs that start with a start codon, end with a stop codon, have a length of a multiple of three, and have no unidentified bases. For genes with more than one CDS, we took the longest CDS that has the above-mentioned properties. A total of 16,627 of these CDSs were expressed in 53 SAGE libraries (see "Computation of gene expression levels" below). For the analysis of each codon, we included only genes in which its corresponding amino acid has at least 10 occurrences.

### Gene copy numbers data

Gene copy number data was taken from the genomic tRNA database, generated by Todd Lowe, using his tRNA-scan program and posted at  (May 2004 assembly). In this data, pseudo-genes have already been removed. We use tRNA gene copy numbers as an assumed estimate of cellular tRNA abundance (see also [12,25]).

### Relative synonymous codon usage (RSCU)

To estimate the codon bias for a single codon we use the *relative synonymous codon usage*, or RSCU, [32] defined as the observed frequency of a codon in the gene *g *divided by the frequency expected if all its synonymous codons are equally frequent, namely,

RSCUig=fig1/syn(i)
 MathType@MTEF@5@5@+=feaafiart1ev1aaatCvAUfKttLearuWrP9MDH5MBPbIqV92AaeXatLxBI9gBaebbnrfifHhDYfgasaacH8akY=wiFfYdH8Gipec8Eeeu0xXdbba9frFj0=OqFfea0dXdd9vqai=hGuQ8kuc9pgc9s8qqaq=dirpe0xb9q8qiLsFr0=vr0=vr0dc8meaabaqaciaacaGaaeqabaqabeGadaaakeaacqqGsbGucqqGtbWucqqGdbWqcqqGvbqvdaqhaaWcbaGaemyAaKgabaGaem4zaCgaaOGaeyypa0ZaaSaaaeaacqWGMbGzdaqhaaWcbaGaemyAaKgabaGaem4zaCgaaaGcbaGaeGymaeJaei4la8Iaem4CamNaemyEaKNaemOBa4MaeiikaGIaemyAaKMaeiykaKcaaaaa@42B1@

where fig
 MathType@MTEF@5@5@+=feaafiart1ev1aaatCvAUfKttLearuWrP9MDH5MBPbIqV92AaeXatLxBI9gBaebbnrfifHhDYfgasaacH8akY=wiFfYdH8Gipec8Eeeu0xXdbba9frFj0=OqFfea0dXdd9vqai=hGuQ8kuc9pgc9s8qqaq=dirpe0xb9q8qiLsFr0=vr0=vr0dc8meaabaqaciaacaGaaeqabaqabeGadaaakeaacqWGMbGzdaqhaaWcbaGaemyAaKgabaGaem4zaCgaaaaa@30E0@ is the frequency of the codon *i*, within its synonymous codon group, in the gene *g*, and syn(*i*) is the degeneracy of the amino acid coded by *i *(the number of synonymous codons for *i*).

In order to eliminate the possible influence of isochoric structures and mutational bias, the RSCU was corrected for background nucleotide content, by replacing 1/*syn*(*i*) with Einc
 MathType@MTEF@5@5@+=feaafiart1ev1aaatCvAUfKttLearuWrP9MDH5MBPbIqV92AaeXatLxBI9gBaebbnrfifHhDYfgasaacH8akY=wiFfYdH8Gipec8Eeeu0xXdbba9frFj0=OqFfea0dXdd9vqai=hGuQ8kuc9pgc9s8qqaq=dirpe0xb9q8qiLsFr0=vr0=vr0dc8meaabaqaciaacaGaaeqabaqabeGadaaakeaacqWGfbqrdaqhaaWcbaGaemyAaKgabaGaemOBa4Maem4yamgaaaaa@31FB@ (*g*), the expected proportion of *i *in *g *calculated according to the non-coding surrounding of the gene:

RSCU′i(g)=figEinc(g)
 MathType@MTEF@5@5@+=feaafiart1ev1aaatCvAUfKttLearuWrP9MDH5MBPbIqV92AaeXatLxBI9gBaebbnrfifHhDYfgasaacH8akY=wiFfYdH8Gipec8Eeeu0xXdbba9frFj0=OqFfea0dXdd9vqai=hGuQ8kuc9pgc9s8qqaq=dirpe0xb9q8qiLsFr0=vr0=vr0dc8meaabaqaciaacaGaaeqabaqabeGadaaakeaacqqGsbGucqqGtbWucqqGdbWqcuqGvbqvgaqbamaaBaaaleaacqqGPbqAaeqaaOGaeiikaGIaem4zaCMaeiykaKIaeyypa0ZaaSaaaeaacqWGMbGzdaqhaaWcbaGaemyAaKgabaGaem4zaCgaaaGcbaGaemyrau0aa0baaSqaaiabdMgaPbqaaiabd6gaUjabdogaJbaakiabcIcaOiabdEgaNjabcMcaPaaaaaa@439C@

Einc
 MathType@MTEF@5@5@+=feaafiart1ev1aaatCvAUfKttLearuWrP9MDH5MBPbIqV92AaeXatLxBI9gBaebbnrfifHhDYfgasaacH8akY=wiFfYdH8Gipec8Eeeu0xXdbba9frFj0=OqFfea0dXdd9vqai=hGuQ8kuc9pgc9s8qqaq=dirpe0xb9q8qiLsFr0=vr0=vr0dc8meaabaqaciaacaGaaeqabaqabeGadaaakeaacqWGfbqrdaqhaaWcbaGaemyAaKgabaGaemOBa4Maem4yamgaaaaa@31FB@(*g*) is computed as in [28]. This method of computing Einc
 MathType@MTEF@5@5@+=feaafiart1ev1aaatCvAUfKttLearuWrP9MDH5MBPbIqV92AaeXatLxBI9gBaebbnrfifHhDYfgasaacH8akY=wiFfYdH8Gipec8Eeeu0xXdbba9frFj0=OqFfea0dXdd9vqai=hGuQ8kuc9pgc9s8qqaq=dirpe0xb9q8qiLsFr0=vr0=vr0dc8meaabaqaciaacaGaaeqabaqabeGadaaakeaacqWGfbqrdaqhaaWcbaGaemyAaKgabaGaemOBa4Maem4yamgaaaaa@31FB@ (*g*) takes into account the background nucleotide, dinucleotide and trinucleotide composition. In addition, as pointed out by Duret and Hurst [33], non-coding DNA is subject to insertion of AT rich transposeable elements. To avoid the possibility that codon usage may be artefactually inflated for G and C ending codons, we masked repetitive elements in non-coding regions which are served for the background correction, using RepeatMasker .

### Major codons

We define a major codon as a codon with the following properties:

1. Its Relative Gene Frequency, or RGF [12] value is greater than 1 (the relative gene frequency of tRNA genes is the observed tRNA-gene copy number in the human genome divided by the frequency expected if all isoacceptor tRNA genes for that amino acid were equally frequent in the genome)

2. A major codon has at least two tRNA genes more than a non-major codon.

3. At most half the codons encoding for a given amino acid are major codons.

By property 1, and the assumed relation between tRNA gene copy numbers and cellular tRNA abundance, a major codon is assumed to have a translational advantage over non-major codons, as it is assumed to have more tRNAs. Thus, the frequency of major codons in a gene can serve as a rough estimate of a gene's translation efficiency. The aim of property 2 is to make this estimate more robust, as the number of tRNA genes is updated with the human genome assemblies, and since the assumed relation between tRNA-gene copy numbers and the frequency of tRNA molecules is by no means accurate.

Table 1 shows the major codons (underlined). As explained below, in order to compute the expected codon frequencies, the amino acids of degeneracy 6 were split into two amino acids of degeneracies 2 and 4 respectively. For example, the two parts of Ser are indicated as Ser2 and Ser4 respectively, and similarly for Arg and Leu. By the above properties Arg2, Arg4, Glu, Leu2, and Lys do not have major codons and are excluded from the analysis.

### Relative major codon usage (RMCU)

While the relative synonymous codon usage (RSCU) is a measure of codon bias for a single codon (see above), the *relative major codon usage *is a measure of major codons frequency for an amino acid. It is defined in a similar manner as RSCU. The RMCU of the amino acid *A *in the gene *g *is the observed proportion of major codons encoding for *A *in *g*, divided by the expected proportion:

RMCUAg=∑fig∑Einc(g)
 MathType@MTEF@5@5@+=feaafiart1ev1aaatCvAUfKttLearuWrP9MDH5MBPbIqV92AaeXatLxBI9gBaebbnrfifHhDYfgasaacH8akY=wiFfYdH8Gipec8Eeeu0xXdbba9frFj0=OqFfea0dXdd9vqai=hGuQ8kuc9pgc9s8qqaq=dirpe0xb9q8qiLsFr0=vr0=vr0dc8meaabaqaciaacaGaaeqabaqabeGadaaakeaacqqGsbGucqqGnbqtcqqGdbWqcqqGvbqvdaqhaaWcbaGaemyqaeeabaGaem4zaCgaaOGaeyypa0ZaaSaaaeaadaaeabqaaiabdAgaMnaaDaaaleaacqWGPbqAaeaacqWGNbWzaaaabeqab0GaeyyeIuoaaOqaamaaqaeabaGaemyrau0aa0baaSqaaiabdMgaPbqaaiabd6gaUjabdogaJbaakiabcIcaOiabdEgaNjabcMcaPaWcbeqab0GaeyyeIuoaaaaaaa@45A2@

where fig
 MathType@MTEF@5@5@+=feaafiart1ev1aaatCvAUfKttLearuWrP9MDH5MBPbIqV92AaeXatLxBI9gBaebbnrfifHhDYfgasaacH8akY=wiFfYdH8Gipec8Eeeu0xXdbba9frFj0=OqFfea0dXdd9vqai=hGuQ8kuc9pgc9s8qqaq=dirpe0xb9q8qiLsFr0=vr0=vr0dc8meaabaqaciaacaGaaeqabaqabeGadaaakeaacqWGMbGzdaqhaaWcbaGaemyAaKgabaGaem4zaCgaaaaa@30E0@ is the frequency of the codon *i *within its synonymous codon group, in the gene *g*, Einc
 MathType@MTEF@5@5@+=feaafiart1ev1aaatCvAUfKttLearuWrP9MDH5MBPbIqV92AaeXatLxBI9gBaebbnrfifHhDYfgasaacH8akY=wiFfYdH8Gipec8Eeeu0xXdbba9frFj0=OqFfea0dXdd9vqai=hGuQ8kuc9pgc9s8qqaq=dirpe0xb9q8qiLsFr0=vr0=vr0dc8meaabaqaciaacaGaaeqabaqabeGadaaakeaacqWGfbqrdaqhaaWcbaGaemyAaKgabaGaemOBa4Maem4yamgaaaaa@31FB@ (*g*) is the expected proportion of *i *in *g *calculated according to the non-coding surrounding of the gene (see above), and both sums are taken over the major codons of encoding for the amino acid *A*.

### Computation of gene expression levels

Expression levels for individual genes were taken from SAGE  on January 5, 2005). Only tags that matched a named gene were taken into account. The dataset was modified to avoid possible GC biases in SAGE [34]; we removed 7 libraries with mean tag GC > 0.5, as in [35]. The resulting SAGE tag/tissue data set was based on 53 libraries representing 20 tissues.

Tag counts in each library were converted to relative values (tags per 200,000) and then averaged over all libraries representing the same tissue type. If a tag was found only once in a tissue type the observation was ignored as a likely sequencing error.

Following [35], we used two measures of expression level: 1) breadth of expression, as the number of tissues in which a gene was expressed, and 2) maximum expression level across tissues. The two methods are highly correlated (R = 0.71, p < < 10^-100^, over all the genes in this analysis).

### Randomization

Our statistical analyses involve large subsets of all known human genes; hence the sample sizes are large. Since low hypothesis tests' p-values can easily be obtained for large random samples, we have to make sure that the small p-values we obtain in our tests indicate non-random phenomena. Thus, each analysis we performed was accompanied by an identical analysis with one of the variables randomly permuted. This randomization was performed 100 times and the minimal p-value over these 100 randomizations was taken. This p-value was compared with the actual p-value obtained for the non-randomized analysis. The hypothesis was accepted as described below.

### t-test analysis

We examined the relation between the RMCU (see above) of each amino acid and the different measures of gene expression (and similarly for the RSCU of each codon). For each amino acid, the relevant genes (the genes where the amino acid in question appears at least 10 times) were divided into 4 bins, according to expression level. To check whether there is a relation between RMCU and expression level we performed a t-test to compare the average RMCU in the group of 25% of the genes with the lowest expression level and the group of the 25% of the genes with the highest expression level. We concluded an ascending (or descending) relation if 1) the p-value obtained for the t-test was lower than the minimal p-value for 100 randomizations and 2) the correlation coefficient was positive (negative) and its p-value was lower than the minimal correlation coefficient p-value for 100 randomizations. For example, the t-test performed for the average RMCU of Glycine in the class of the 25% lowly expressed genes and the class of 25% highly expressed genes (calculated by breadth of expression) yielded a p-value of 1.2 × 10^-5^, and the correlation coefficient was R = 0.07 with a p-value of 10^-13^. The corresponding minimal random p-values were 0.014 and 0.033 respectively. Thus, we concluded an ascending relation between RMCU of Glycine and breadth of expression. This relation is evident from Figure 1.

## Authors' contributions

DK conceived of the study and coordinated it, designed the analysis, performed the statistical analysis, interpreted the results, and wrote the manuscript. YL conceived of the study and coordinated it, designed the analysis, interpreted the results, and wrote the manuscript.

## Figures and Tables

**Table 1:** Relation between RSCU' and measures of expression for all codons of amino acids of degeneracy greater than one. For each codon, only the genes in which the corresponding amino acid has a frequency of at least 10 were considered. Major codons are underlined. ^w ^indicates G/U wobble (by Lander et al. 2001). * indicates translationally weak codons. The (+) sign indicates that the average RSCU' in class of 25% lowly expressed genes is significantly lower than the average RSCU' in class of 25% highly expressed genes, while the (-) sign represents the opposite. N.S. indicates that the RSCU' is not significantly different

A	B	C	D	E
Amino acid	Codon	tRNA GCN	RSCU vs maximum expression	RSCU vs breadth of expression

Ala	GCA	9	N.S.	+
	GCC^W^	0	N.S.	-
	GCG*	5	-	-
	GCT	29	+	+
Arg2	AGA	6	N.S.	+
	AGG	5	-	-
Arg4	CGA	6	N.S.	+
	CGC^W^	0	N.S.	-
	CGG	5	N.S.	N.S.
	CGT	7	N.S.	N.S.
Asn	AAC	28	N.S.	-
	AAT^W^	1	N.S.	N.S.
Asp	GAC	18	-	-
	GAT^W^	0	+	+
Cys	TGC	30	N.S.	N.S.
	TGT^W^	0	N.S.	N.S.
Gln	CAA*	11	-	-
	CAG	21	+	+
Glu	GAA	12	N.S.	+
	GAG	13	N.S.	-
Gly	GGA	9	N.S.	+
	GGC	15	+	N.S.
	GGG*	7	-	-
	GGT^W^	0	+	+
His	CAC	11	N.S.	N.S.
	CAT^W^	0	N.S.	N.S.
Ile	ATA*	5	-	-
	ATC^W^	5	+	N.S.
	ATT	14	N.S.	+
Leu2	TTA	7	-	N.S.
	TTG	6	N.S.	N.S.
Leu4	CTA*	3	-	N.S.
	CTC^W^	0	-	-
	CTG	10	+	N.S.
	CTT	12	-	N.S.
Lys	AAA	17	-	N.S.
	AAG	17	N.S.	N.S.
Phe	TTC	12	N.S.	N.S.
	TTT^W^	0	N.S.	+
Pro	CCA	7	+	+
	CCC^W^	0	-	-
	CCG*	4	-	-
	CCT	10	N.S.	+
Ser2	AGC	8	N.S.	N.S.
	AGT^W^	0	N.S.	+
Ser4	TCA	5	N.S.	+
	TCC^W^	0	-	-
	TCG*	4	-	-
	TCT	11	+	+
Thr	ACA	8	N.S.	+
	ACC^W^	0	N.S.	-
	ACG*	6	-	-
	ACT	10	N.S.	N.S.
Tyr	TAC	14	N.S.	-
	TAT^W^	1	N.S.	+
Val	GTA*	5	N.S.	+
	GTC^W^	0	N.S.	N.S.
	GTG	16	-	-
	GTT	11	N.S.	+

**Table 2:** RMCU vs. maximum expression and breadth of expression for amino acids with major codons. The (+) sign indicates significant increase. The (-) sign indicates significant decrease, and N.S. indicates non-significant tendency

Amino acid	RMCU vs. maximum expression	RMCU vs. breadth of expression
Ala	+	+
Asn	N.S.	-
Asp	-	-
Cys	N.S.	N.S.
Gln	+	+
Gly	+	+
His	N.S.	N.S.
Ile	N.S.	+
Leu4	+	+
Phe	N.S.	N.S.
Pro	N.S.	+
Ser2	N.S.	N.S.
Ser4	+	+
Thr	N.S.	+
Tyr	N.S.	-
Val	N.S.	N.S.

**Table 3:** Frequency (Columns A-C), Dufton's (1997) size/complexity score [29] (Columns D-F) and Trifonov (2004) chronology ranking [30] (Columns G-I) of amino acids of degeneracy greater than one that have major codons. (In [30] there is no ranking for the two-fold part of Ser (Ser2), but its initiation was placed somewhere between Thr and Ile.)

A	B	C	D	E	F	G	H	I
		
Amino acid	Group	Frequency per 10,000 codons	Amino acid	Group	Size/complexity score	Amino acid	Group	Chronology ranking
		
Leu4	A	786	Gly	A	1	Gly	A	3.5
Ala	A	704	Ala	A	4.76	Ala	A	4
Gly	A	663	Val	B	12.28	Val	B	6
Pro	A	628	Ile	A	16.04	Asp	B	6.3
Val	B	598	Leu4	A	16.04	Ser4	A	7.3
Thr	A	526	Ser2	B	17.86	Pro	A	7.6
Ser4	A	500	Ser4	A	17.86	Leu4	A	9.4
Asp	B	479	Thr	A	21.62	Thr	A	9.9
Gln	A	477	Pro	A	31.8	Ser2	B	
Ile	A	433	Asp	B	32.72	Ile	A	11
Phe	B	365	Asn	B	33.72	Asn	B	11.3
Asn	B	362	Gln	A	37.48	Gln	A	11.4
Ser2	B	326	Phe	B	44	His	B	13.3
Tyr	B	266	Tyr	B	57	Phe	B	13.8
His	B	261	Cys	B	57.16	Cys	B	14.2
Cys	B	227	His	B	58.7	Tyr	B	15.2
